# Coumarin-Encapsulated Solid Lipid Nanoparticles as an Effective Therapy against Methicillin-Resistant *Staphylococcus aureus*

**DOI:** 10.3390/bioengineering9100484

**Published:** 2022-09-20

**Authors:** Mohammed H. Alqarni, Ahmed I. Foudah, Aftab Alam, Mohammad A. Salkini, Magdy M. Muharram, Nikolaos E. Labrou, Pinki Rawat

**Affiliations:** 1 Department of Pharmacognosy, College of Pharmacy, Prince Sattam Bin Abdulaziz University, Alkharj 11942, Saudi Arabia; 2 Department of Microbiology, College of Science, Al-Azhar University, Nasr City 11884, Cairo, Egypt; 3 Laboratory of Enzyme Technology, Department of Biotechnology, School of Food, Biotechnology and Development, Agricultural University of Athens, 75 Iera Odos Street, GR-11855 Athens, Greece; 4 Maharana Pratap College of Pharmacy, Abdul Kalam Technical University, Kanpur 209217, Uttar Pradesh, India

**Keywords:** bacterial infections, *Staphylococcus aureus*, methicillin-resistant *Staphylococcus aureus*, coumarin, solid lipid nanoparticles

## Abstract

Bacterial infections caused by antibiotic-resistant pathogens are a significant public health problem. This is because the transmission of infectious diseases is shifting, and new antibiotic-resistant strains of bacteria are emerging. The development of biofilms that are resistant to antibiotics poses another hurdle to drugs and treatment alternatives. Therefore, there is an urgent need to develop innovative strategies to effectively eliminate antibiotic-resistant microorganisms effectively. Natural coumarins have broad spectrum bioactivity and the potential for lower resistance. Coumarin is a secondary metabolite found in certain plants, fungi, and bacteria. It is highly effective against methicillin-resistant *Staphylococcus aureus* (MRSA). Therefore, coumarin can be used as an alternative to combat MRSA. However, most antibacterial agents lack selective targeting of pathological sites, limiting the efficacy of their antibacterial activity. Efficient MRSA treatments can be achieved through nanoparticle (NPs)-based targeted therapies. To address this challenge, a novel coumarin-loaded solid lipid nanocarrier for MRSA was developed to overcome this challenge. The developed systems exhibited a particle size of 138.5 ± 76.06 nm and a polydispersity index (PDI) of 0.245 ± 0.00. The zeta potential of coumarin-loaded SLNs was reported to be −22.2 ± 8.15 mV with a spherical shape. The encapsulation efficiency of coumarin was reported to be 63.09 ± 3.46% in the final formulation. The developed formulation was biocompatible with a minimum inhibitory concentration (MIC) of 1.08 µg/mL. This study suggests that coumarin-loaded SLNs can effectively treat MRSA infections.

## 1. Introduction

Eliminating infections caused by pathogens associated with biofilms is challenging [1]. These infections can, therefore, progress from the acute to the chronic phase and cause severe complications [2]. The growth of bacteria in biofilms leads to several chronic infections, mainly characterized by persistent inflammation and tissue damage [3]. The World Health Organization (WHO) has named resistant bacteria as one of the world’s top 10 research priorities. It is estimated that up to 7 million people worldwide die each year from antibiotic resistance. If no effective therapy is found, the death rate could rise to 10 million per year by 2050 [4].

Bacterial biofilms epitomize a substantial problem in medicine. In contrast, biofilms are ubiquitous life forms that can seriously complicate the situation in various settings, including healthcare, medical facilities, and biomedical products such as medical implants [5]. Biofilm development is strongly favored by several microbes, including *Pseudomonas aeruginosa*, *Staphylococcus epidermidis*, *Staphylococcus aureus*, and *Escherichia coli* [3]. The exact mechanisms of the onset of diseases caused by microorganisms associated with biofilms are not well understood. However, studies indicate cell separation, endotoxin production, and host immune system resistance are also involved [6].

Biofilm formation is a characteristic feature of *Staphylococcus aureus* (*S. aureus*) and *S. epidermidis* infection. It is composed of several layers of bacteria covered in exo-polysaccharide glycocalyx. [7]. *S. aureus* is an important human pathogen responsible for more than 40 percent of infections in intensive care units. Today, powerful antibiotics such as vancomycin and linezolid are used to treat MRSA. The main problem with these antibiotics is the development of resistant strains [2]. Multidrug-resistant microorganisms have emerged due to antibiotic overuse (MDR). In the past few years, antibiotic-resistant *S. aureus* has been a cause for concern. Methicillin-resistant *S. aureus* has arisen due to increased resistance (MRSA). MRSA is one of the most common antibiotic-resistant pathogens that can spread between humans and animals and seriously threaten human health [8]. MRSA infects various organs and tissues, which can lead to various diseases, including endocarditis, chronic osteomyelitis, pneumonia, and bacteremia [9,10]. Therefore, research community contributions are needed to develop more effective treatments for MRSA bacterial infections, skin infections, and other infections that are more common and deadly [11].

All these concerns have paved the approach for pondering effective treatment approaches. New anti-MRSA drugs with higher efficacy and lower cytotoxicity are urgently needed to treat infections caused by such resistant strains [12]. Coumarin is a secondary metabolite of certain plants, fungi, and bacteria [13]. Recently, the results of different research groups have indicated that coumarin has potent activity against MRSA [14,15,16,17,18]. For instance, recently, Qu et al. proclaimed that the coumarin derivative 3,3′-(3,4-dichlorobenzylidene)-bis-(4-hydroxycoumarin) (abbreviated DCH) is enormously efficacious against MRSA [17].

To enhance the therapeutic benefits of coumarin against MRSA infections, a novel delivery system needs to be developed. Solid lipid nanoparticles (SLNs) are an excellent alternative to enhance the therapeutic effects of coumarin. SLNs have been studied as biodegradable transport systems for many purposes [19,20,21,22]. In the last two decades, SLNs have been widely used in the pharmaceutical industries as a delivery system for polymer nanoparticles, liposomes, and emulsions. The benefits of using SLN as a delivery vehicle include increasing the physical-chemical stability of the core material. The combination of capsules reduces the leakage of active drugs, encapsulated compounds, and interactions with the emulsifier coat. SLNs offer a controlled drug-release pattern with maximum drug encapsulation [23]. Hence, ion pairing of coumarin with stearic acid and Tween^®^ 20, which exhibits antibacterial activity, could be used as a technique to simultaneously improve encapsulation in SLNs and enhance the antibacterial activity of this nanocarrier system. The objective of this study was achieved from the newly developed delivery system.

## 2. Materials and Methods

### 2.1. Materials

The coumarin was purchased by Saudi Arabia’s Koch Light Laboratory Limited. Stearic acid, polysorbate 20 (Tween^®^ 20), and dialysis bags (molecular weight of 14,000 Da) were purchased from Sigma-Aldrich. All chemicals were of analytical grade and did not require further purification before use. Deionized (DI) water was used to prepare all solutions.

### 2.2. Preparation of Tween^®^ 20 Stabilized Coumarin-Loaded SLNs

The SLN loaded with coumarin was synthesized according to the methods of Affram et al. 2020 [24] and Shah et al. 2022 [25]. The formulation of SLN loaded with coumarin was prepared using high-pressure cold homogenization technology. A short measure of the quantity of stearic acid (100 mg) was melted on a hot plate at 75–80 °C, and 20 mg of coumarin was added. Then, they were mixed in another Tween^®^ 20 beaker (150 μL) and 15 mL of water. The hot microemulsion o/w was immediately dispersed into cold ice water (50 mL, 2–4 °C) and intermittently homogenized (10,000 rpm) to generate SLN dispersions.

### 2.3. Characterization of Prepared Nanoparticles

#### 2.3.1. Particle Size, Polydispersity Index, and Zeta Potential Determination

The measurement of particle size and electrophoretic mobility was carried out by dynamic light diffusion (DLS). The ZS Zetasizer was used to determine the potential for the ZS and the formulation of the drug (Malvern Instrument, Malvern, UK) [2]. Before the measurements, the SLN dispersion was properly diluted with ultra-purified water to eliminate multiple dispersion and the effects of concentration of the composition on viscosity. The results are presented as an average value of the water-dynamic diameter and polydispersity index (P.D.I.) of intensity weight. The ZETA potential was calculated using Zeta by electrophoretic motion [26].

#### 2.3.2. Estimation of Encapsulation Efficiency (% EE)

Encapsulation efficiency (EE) refers to the rate at which the weight of the drug is encapsulated in the transport system and the total amount of the added drug. To determine EE percentages, free and trapped drugs were measured. The SLN dispersion of 1 mL loaded coumarin was centrifuged for 10 min with a Remi centrifuge at 5000 rpm ambient temperature. The supernatant was separated without disturbing the pellet of the drug in the sediment (free drug). Then, 4.5 mL of methanol was used for its dilution. In addition, it was well mixed to facilitate the complete extraction of the drug from lipid to methanol. The drug was analyzed at 360 nm with UV—a spectrophotometer against methanol as a blank. The % EE was designed and established with Equation (1).

(1) $$\%\;\mathrm{EE}=\frac{\left(Total\;amount\;of\;coumarin-Amount\;of\;coumarin\;in\;\mathrm{supernatant}\right)}{Total\;amount\;ofcoumarin}\times 100$$

#### 2.3.3. Morphology

The surface morphology and shape of the newly synthesized coumarin-loaded SLN dispersion were evaluated by a field emission-scanning electron microscope (FE-SEM; ZEISS, Ultra Plus, Wetzlar, Germany).

#### 2.3.4. Cell Toxicity Assay

To carry out the cell toxicity assay, we followed the same procedure as the MTT assay published by Kalhapure et al. [27]. In this procedure, first, we seeded the cells equivalent to 2.5 × 10^3^. It was cultured at 37 °C for 24 h. After this, an appropriate amount of coumarin-loaded SLN dispersion in a concentration ranging from 20–100 μg/mL was added to selected basal epithelial cell (A549) and embryonic kidney cell (HEK-293) cell lines for evaluation. After this, it was kept in incubation for 48 h. After the incubation period ended, the culture medium was replaced by fresh media (100 μL) and PBS (5 mg/mL) MTT solution (100 μL). After adding the MTT solution, it was kept for incubation at 37 °C for 4 h. After completion, the reaction was stopped by injecting cells into 100 μL DMSO at a wavelength of 540 nm; the optical density of each well was evaluated using a microplate spectrophotometer. The percentage cell viability was calculated as follows:

(2) $$\%\mathrm{Cell}\;\mathrm{viability}=\frac{\left(A540\;\mathrm{nm}\;\mathrm{treated}\;\mathrm{cells}\right)}{\left(A540\;nm\;untreated\;cells\right)}\times 100\ldots \ldots$$

#### 2.3.5. In Vitro Drug Release

In vitro release of SLNs loaded with coumarin was performed using the previously described washing bag technique [2]. The experiment was performed at 37 °C for 48 h in release media containing phosphate buffer saline (PBS; 100 mM, pH 7.4). The release medium was fortified with 1% Tween 80 to accelerate the dissolution of the drug. A dialysis membrane with a molecular weight cut-off of 14,000 Da was used in the study. Before use, the dialysis bag was soaked in distilled water for 12 h. A 5 mL aliquot of the prepared SLN formulation was placed in a dialysis bag, then the bag was sealed and immersed in 50 mL of prewarmed release medium at (37 ± 0.5 °C) in clear glass bottles. To maintain a septic condition, 1 mL of the release medium was withdrawn at a predetermined time period and immediately replaced with the same volume of fresh medium. To determine how much coumarin was present in the sample, the scientists used UV-vis spectroscopy, which has a maximum detection limit of 360 nm. Triplicate examinations were performed.

#### 2.3.6. In Vitro Antibacterial Activity

The broth microdilution method was used to test antibacterial activity in BAA-1683 strains (MRSA), and the results were evaluated according to previously established procedures [28,29]. To carry this out, a stock solution of SLNs loaded with coumarin and pure coumarin was prepared in water to ensure complete solubility at a concentration of 1 mg/mL. Then, 100 µL of the nutritional broth and the saboraud dextrose broth were dispensed from well 1 to well 10. The coumarin-loaded SLNs and coumarin powder (100 µL) were added to the first well. The solution was diluted in series from 1 to 10 L, while 100 µL from 10 L was discarded. Then, 100 µL bacterial suspension was added to all dilutions from 1 to 10. The plate was incubated at 37 °C for 24 h. After incubation, each well was measured by ELISA readers (Erba) with wavelengths of 640 nm. The above procedure applied to all microbial strains. All microorganisms determined the concentrations of samples and standards that inhibited 50% growth of bacteria. The procedure was repeated every three days and all MIC results. Studies were conducted three times. Blank dextran formulations were used as negative controls, while coumarin formulations were used as positive controls.

#### 2.3.7. Bactericidal Time-Kill Kinetics

We performed bactericidal time-kill kinetics using the CLSI M26-A guidelines [29]. An MRSA culture in Mueller–Hinton broth (MHB) was diluted with phosphate buffer to 5 × 10^5^ CFU/mL. The SLNs loaded with coumarin powder and coumarin were added at a concentration five times greater than the MIC results. Sterile water was added to the test samples and used as a negative control. The effectiveness of bacterial cells was monitored continuously for 24 h. The samples were collected at specified intervals, diluted serially in PBS, and deposited in triplicate on Mueller–Hinton agar (MHA) plates. The incubation was carried out overnight at 37 °C. After incubation, the bacteria’s development was estimated with colonies on the plates.

## 3. Results

### 3.1. Particle Size, Polydispersity Index (PDI), and Zeta Potential Determination

**Figure 1:**
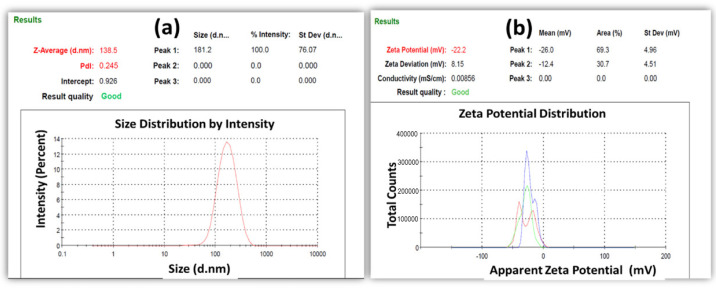
(**a**) Particle sizes and polydispersity index (**b**) Zeta potential of coumarin-loaded SLN dispersions (Three colored lines represent that the same zeta potential was taken three times, and these colors is computer generated.)

Particle size is a crucial technical parameter that confirms the development of nanoparticles. Particle size and size distribution are important factors in assessing the stability of the dosage form during storage. Figure 1a shows the average size and PDI of a coumarin-loaded SLN. The formulation of coumarin-loaded SLNs achieved an average particle size of 138.5 ± 76.06 nm and a PDI of 0.245 ± 0.00. The zeta potentials of the coumarin-loaded SLNs were reported to be −22.2 ± 8.15 mV. The strong negative surface charge determined when measuring the zeta potential of nanoparticles indicated electrostatic repulsion between the particles. It is an essential indicator of stability. This conclusion agrees with Shah et al., who also reported a negative absolute value of zeta potential of SLNs functionalized with drugs [25].

### 3.2. Estimation of Encapsulation Efficiency (% EE) and Morphology

**Figure 2:**
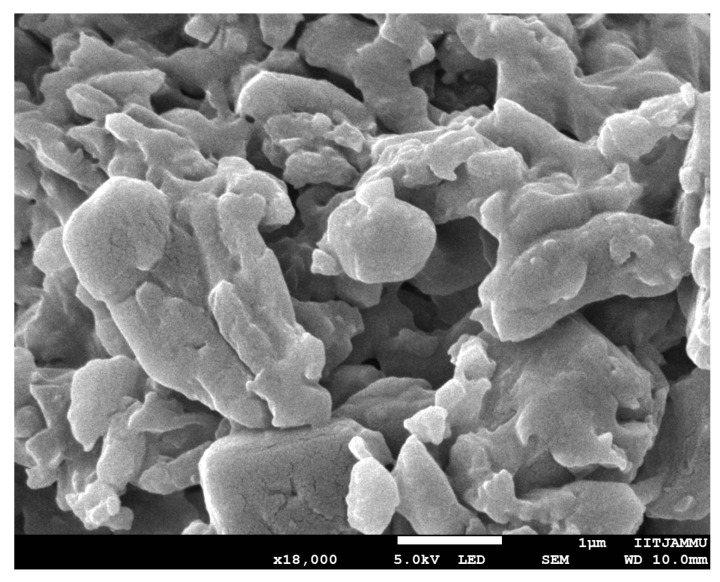
SEM image of coumarin-loaded SLN dispersions.

The composition of the lipid core and the drug material affected the extent of the coumarin capture in SLNs. The encapsulation efficiency of coumarin was found to be 63.09 ± 3.46% in the final formulation. The morphology of the prepared formula was spherical with rough or irregular surfaces by both SEM, as shown in Figure 2. The aggregates may be due to a short time of re-evaporation after centrifugation and drying at ambient temperature.

### 3.3. Cell Toxicity Assay

**Figure 3:**
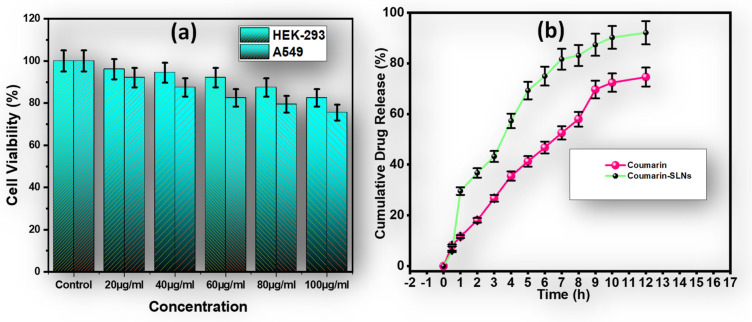
(**a**) The % cell viability assay of coumarin-loaded SLN dispersions in different concentrations; (**b**) the comparative in vitro drug release study of coumarin powder and coumarin-SLNs.

Figure 3a depicts the percentage cell viability of coumarin-loaded SLN dispersions in selected cell lines in different concentrations. The MTT assay showed that more than 75% of the cells were viable in all concentrations. There was no dose-dependent effect on the survival of the cells in any of the cell lines employed for this investigation. There were no significant variations in cell viability between coumarin-loaded SLNs and the control group.

### 3.4. In Vitro Drug Release

The drug release from coumarin-loaded SLN dispersions and pure coumarin is shown in Figure 3b. The in vitro release of SLNs from nanoparticles loaded with coumarin and pure coumarin was studied using the in vitro dialysis bag method in PBS; 100 mM, pH 7.4. This is the only method described in the literature to estimate the release of SLN drugs. Here, we observed that the drug released from pure coumarin was found to be 41.26 ± 2.06 %, whereas in the case of coumarin-SLNs, it was found to be 70.23 ± 3.51% within 5 h. After the completion of 12 h, the drug released was found to be 74.55 ± 3.74% (coumarin) and 92.02 ± 4.60 (coumarin-SLNs). These results suggest that in the case of pure coumarin, the burst release of the drug was observed. This shows the possibility of passing the molecule from the dialysis membrane. On the other hand, a biphasic drug-release profile was observed for the coumarin-loaded SLN dispersion. It can be characterized by an initial burst release within the first two hours followed by a prolonged release. The initial bursting of coumarin can be attributed to the rapid incorporation of coumarin into the shell. The coumarin charged in the SLN core is gradually displaced by two mechanisms: dissolution and diffusion. Similar results and interpretations were reported by Shah et al [26]. These results indicate that coumarin incorporated into SLNs are likely associated with SLNs.

### 3.5. In Vitro Antibacterial Activity 

#### 3.5.1. Minimum Inhibitory Concentration

Furthermore, the antimicrobial effectiveness of SLNs loaded with coumarin was determined. The antimicrobial efficacy of coumarin, the SLN suspension, and coumarin-loaded SLNs were evaluated by calculating the MIC against MRSA. The results are presented in Table 1. The MIC of coumarin was 3.92 μg/mL and 4.93 μg/mL. Interestingly, the MIC of coumarin-loaded SLNs was 1.08 µg/mL and 2.13 µg/mL. This indicated that the coumarin-loaded SLNs have good antimicrobial activity compared to coumarin powder.

#### 3.5.2. Bactericidal Time-Kill Kinetics

**Figure 4:**
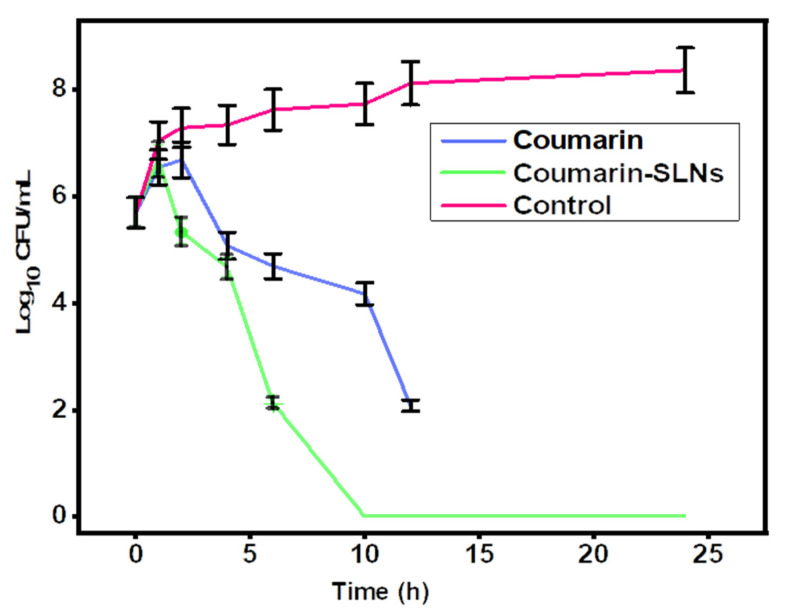
Comparative bactericidal time-kill kinetics of control, coumarin, and coumarin-loaded SLN treatment against MRSA.

Figure 4 shows the microbiological mortality rate of coumarin and SLN loaded with coumarin. We chose five times the MIC of each treatment and incubated the sample for 24 h at 37 °C. Compared to coumarin alone and coumarin-loaded SLNs, bacterial effects were faster, and three logarithms were reduced after 8 h. Then, after 24 h, the bacteria began to kill. The mortality rate caused by coumarin was comparable to that recorded in the scientific literature. This could lead to faster clearance of bacteria in the blood, reducing the duration of treatment and the amount of drug required for effective therapy.

## 4. Discussion

MRSA is the leading cause of morbidity worldwide. In this study, we introduced coumarin into SLN carriers to overcome water-soluble limitations and enhance the therapeutic effect of MRSA bacteria. To this end, we investigated the therapeutic effect of the local administration of coumarin. The results show that coumarin-loaded SLNs release high coumarin concentrations over a long period of time, promoting the reconstruction of new bone and providing effective local antimicrobial activity in cells. All the above results suggest that SLNs may be a promising coumarin drug carrier and further facilitate their development for the delivery of topical, oral, and/or nasal drugs. Our research shows that SLN carriers with coumarin are an effective treatment for MRSA. The strategy is a safe and effective formulation for the treatment of MRSA that provides a moist environment, prevents bacterial infections, and increases patient compliance.

## Figures and Tables

**Table 1:** Comparative minimum inhibitory concentration.

Compound	MIC (µg/mL)	
	24 h	48 h
Vancomycin HCl	2.04	NA
SLN suspension	NA	NA
Coumarin	3.92	4.93
Coumarin-loaded SLNs	1.08	2.13

## Data Availability

Data that support the findings are available within the manuscript.
